# Supplementary material for: ‘You get looked at like you’re failing’: A reflexive thematic analysis of experiences of mental health and wellbeing support for NHS staff
Source: J Health Psychol. Author manuscript; Available in PMC 2023 Aug 1. (PMC10387714; doi:10.1177/13591053221140255)
Supplement: Supplementary Material [file EMS181738-supplement-Supplementary_Material.docx]

# **Appendix 1** – Semi-structured Interview Schedule

# Schedule for participants groups 1 and 2

Group 1: participants who used support interventions and found them helpful
Group 2: participants who used support interventions and found them not helpful or actively unhelpful

## Introduction

This interview is about your experience of the various support services provided to you by your employer. So, we are interested in any staff support or wellbeing programmes or services that have been made available to you. These might be local services or ones that have been available nationally which you could have used to help cope with the demands of the COVID-19 pandemic. We are interested in either programmes or services that were in place prior to March 2020 or new ones put in place since. Examples include helplines, wellbeing activities like mindfulness, or relaxation spaces or coaching.

Could you list any support services at your workplace that you’ve heard of and which you have used?

*(If a participant has used more than one service, prompt them to reflect on each one in turn throughout interview.****)***

## Understanding support use

(*Ask questions 1 and 2 about each support service in turn in order to establish a timeline of usage; when participant started/stopped services, what lead to starting/stopping usage*)

1. For each of the support services you have told me about can you tell me a little more about?

- Who is the service for? (teams, individuals, certain staff groups)
- What kind of support does if offer provide? Put another way, what is the support supposed to do?

1. Can you tell me about the timing of when you have used this/these support service(s)?

(*Aim to establish a timeline of usage; when participant started/stopped* services*, what lead to starting/stopping usage.*)

- What led to you trying this/these support(s)?
- How often do you use it/them?
- Do you use it/them when you’re feeling distressed, sad or anxious, or to prevent these feelings?
- Have there been periods when you’ve used it/them more or less?
- If you have stopped using it/them, what lead to you stopping use (such as lack of time, not supported by line manager)?

1. Has your use of support services or support needs changed since the start of the COVID-19 pandemic?

(*Reflect using information gathered from asking about timeline.*)

- Do you use workplace support more or less?
- Have you started using any new services?
- Have services been more or less useful?
- Have services felt more or less accessible?
- Are you aware of any new services at your workplace, or changes to existing services since the start of the pandemic?

## Benefits of support

1. Thinking about the support service(s) that you have used, do you feel that it has/they have made any difference to you?

| If helpful: | If unhelpful: |
| --- | --- |
| - What kind of impact has it/have they had on your wellbeing/ability to cope with work? - What is it about the service(s) that is/are most helpful? - Do they seem as though they might be helpful to your colleagues? | - What kind of impact has it/have they had on your wellbeing/ability to cope with work? - What is it about the service(s) that is/are most unhelpful? - Do they seem as though they might be helpful to your colleagues? |

1. Do you feel as though this/these support service(s) you’re aware of was/were created with staff like you (your role, your needs) in mind?

- Can you tell me why it feels that way?
- What elements of it are particularly suited or not suited to your needs?

1. Thinking about what we’ve just talked about, how do you think this/these support service(s) could be improved to provide better help, if at all?
2. Are there any support services not currently provided by your workplace that you would like to be provided, either for yourself or for your team?

- Are there any services that you’ve heard of being provided by other Trusts that you think would be useful here?
- Is there any informal support available at work that would be helpful if offered in a formalized or structured way?
- What help would these services provide that isn’t currently available to you?
- How could they be structured so that they are easy to access?

## Access to support

1. How do you hear about available support in your workplace?

- What are the best ways for your workplace to tell you about available services?
- What are the worst ways for your workplace to tell you about available services?
- Do other members of staff seem to be aware of what’s available?

1. How easy or difficult is it to use the support interventions at your workplace?*

- What, if anything, makes them easier to use?
- What, if anything, makes them harder to use?
- How does their use fit into your routine?
- Do you feel like you could use these resources whenever you needed to?
- Do you feel that there is any stigma around using support services?
- Are there services that you feel aren’t available to you because of your role?

1. What practices, if any, are in place that help you access support at work?*

- How do line managers or senior staff make it easier for you to access to support, if at all?
- Are there any practical things, like phones or private spaces, provided to you?

1. If there were support services you heard about but did not access, can you tell me what lead to you choosing not to use them?

(*If participant mentioned any at the beginning of the interview, reference as prompts here.*)

- How easy or difficult to access did they seem?
- How helpful or unhelpful did they seem?

1. Thinking about what we’ve just talked about, how could support services at your workplace be made more accessible, if at all?

## Alternative support

1. Is there any support you get at your workplace that isn’t formally offed by the Trust, such as peer support or informal supervision?

If YES

- Can you describe this support to me?
- How does this support make a difference to you?
- Could this support be encouraged or formalised by the Trust? (If so, how?)
- Does this meet your needs differently to the formal support services on offer (if so, how)?
- Is there anything that you do to encourage peer support within your team?

If NO

- What are your relationships like with your peers/colleagues/managers?
- Would you like more informal support from peers/colleagues/managers?
- Could this support be encouraged or formalised by the Trust? (If so, how?)

1. Can you tell me about any support services you might have accessed outside of work (such as support from GP services or charities)?

- What led to you choosing to use this/these service(s)?
- How does it/do they differ from the interventions provided at work?
- Would it make a difference to you if any of these services were provided by your workplace?

## Interview ending

Is there anything that we haven’t yet covered that you feel is important to tell me?

How did you find the interview?

Thank you for you for talking to me today. I really appreciate your time, and the information you’ve given me today will help us to understand how Trusts can best support staff like yourself. In the same way that we occasionally contact you with updates about how the NHS CHECK survey is doing, we will email you later in the year to update you on our progress and to tell you about the key findings from our study.

We’ll send your £25 gift voucher to the email address that we hold for you within 10 days, unless you’d like to provide me with a different email address that you’d prefer me to send it to. If you have any further comments or questions, you can email me at the nhscheck@kcl.ac.uk email address.

# Schedule for participants groups 3 and 4

Group 3: participants who had heard about support interventions available, but chose not to use them
Group 4: participants who had not heard of available support interventions

## Introduction

*All participants*:

This interview is about your opinion of the various support services provided to you by your employer. So, we are interested in any staff support or wellbeing programmes or services that have been made available to you. These might be local services or ones that have been available nationally which you could have used to help cope with the demands of the COVID-19 pandemic. We are interested in either programmes or services that were in place prior to March 2020 or new ones put in place since. Examples include helplines, wellbeing activities like mindfulness, or relaxation spaces or coaching.

Could you list any support services at your workplace that you’ve heard of, if any?

*(If a participant has heard of more than one intervention, prompt them to reflect on each one throughout interview.****)***

## Usage of support

| *Aware of service(s)*: | *Unaware of services*: |
| --- | --- |
| 1. Can you tell me more about the support service(s) that you have heard of?  - Who is the service for? (teams, individuals, certain staff groups) - What kind of support does it provide? Put another way, what is it supposed to do? - Are you aware of any changes to available services at your workplace since the start of the pandemic? | n/a |

1. Do you feel as though your support needs have changed since the start of the COVID-19 pandemic?

- (*if changed*) What were your needs before and how have they changed?
- (*if not changed*) What were your needs before and how have they continued to be met?
- Are you aware of any new services at your workplace, or changes to existing interventions, since the start of the pandemic?
- Have there been periods during the pandemic when you’ve felt like you would need support more or less?

## Benefits of support

1. In what circumstances might a support service at work be particularly helpful to you or your team?

- What might lead you to using a support service?
- Are there periods of time when support would be particularly helpful?

| *Aware of service(s)*: | *Unaware of services*: |
| --- | --- |
| 1. What led to you choosing not to use any of the services available at your workplace?  - How helpful or unhelpful did they seem? - Does it feel as though they might be useful to your colleagues?  1. Do you feel as though these support services were created with staff like you (your role, your needs) in mind?  - Can you tell me why it feels that way? - What particular needs should be considered?  1. How do you think these support services could be improved to better meet your needs?  - What impact would you like an intervention to have? - What elements of it/them could be made to be more helpful? | n/a  n/a  n/a |

1. Are there any support services not currently provided by your workplace that you would like to be provided, either for yourself or for your team?

- Are there any services that you’ve heard being provided by other Trusts that you think would be useful here?
- What help would these interventions provide that isn’t currently available to you?
- How could they be structured so that they were easy to access?

## Access to support

| *Aware of service(s)*: | *Unaware of service(s)*: |
| --- | --- |
| 1. How do you hear about available support in your workplace?  - What are the best ways for your workplace to tell you about available services? - What are the worst ways for your workplace to tell you about available services? - Do other members of staff seem to be aware of what’s available?  1. How easy or difficult do you think it would be to use the support services at your workplace?  - What would make them easier to use? - What would make them harder to use? - How would using them fit into your routine? - Do you feel that there is any stigma around using support in your workplace? - Do you feel like you could use support services whenever you needed to? - Are there interventions that you feel aren’t available to you because of your role?  1. What, if any, practices are in place that help you access support at work should you wish to use it?  - How do line managers or senior staff make it easier for staff to access to support, if at all? - Are there any practical things, like phones or private spaces, provided to you?  1. Thinking about what we’ve just talked about, how could support interventions at your workplace be made more accessible, if at all? | What would be the best and worst ways for your workplace to tell you about available support interventions?  How easy or difficult do you think it would be to use a support intervention at work, such as [*examples of services available in their Trust*]?   - What would make them easier to use? - What would make them harder to use? - How could a service be designed so that it could fit into your routine or workday? - Do you feel that there is any stigma around using support services in your workplace?   What practices could be put in place at your workplace to help you access support services?   - How could line managers or senior staff make it easier for staff access to support? - Are there any practical things, like phones or private spaces, provided to you?   n/a |

## Alternative support

1. Is there any support you get at your workplace that isn’t formally offed by the Trust, such as peer support or informal supervision?

If YES

- Can you describe this support to me?
- How does this support make a difference to you?
- Could this support be encouraged or formalised by the Trust? (If so, how?)
- Does this meet your needs differently to the formal support services on offer (if so, how)?

If NO

- What are your relationships like with your peers/colleagues/managers?
- Would you like more informal support from peers/colleagues/managers?
- Could this support be encouraged or formalised by the Trust? (If so, how?)

1. Can you tell me about any support services you might have accessed outside of work (such as support from GP services or charities)?

- What led to you choosing to use this/these service(s)?
- How does it/do they differ from the services provided at work?
- Does it feel like these interventions fully meet your needs, or would support at work be useful in addition?

## Interview ending

Is there anything that we haven’t yet covered that you feel is important to tell me?

How did you find the interview?

Thank you for you for talking to me today. I really appreciate your time, and the information you’ve given me today will help us to understand how Trusts can best support staff like yourself. In the same way that we occasionally contact you with updates about how the NHS CHECK survey is doing, we will email you later in the year to update you on our progress and to tell you about the key findings from our study.

# **Appendix 2** – Participant characteristics and services used

Table 1: *Age, sex, and role* *characteristics of sample, and knowledge/use of services*

| **Characteristic** |  | **N (%)** |
| --- | --- | --- |
| Age |  | Mean = 43; SD = 10.95 |
| Sex | Male | 20 (42) |
|  | Female | 28 (58) |
| Primary role | Clinical | 25 (52) |
|  | Non-clinical | 23 (48) |
| Knowledge of services | Aware of at least one available support service | 48 (100) |
|  | Unaware of any available support services | 0 (0) |
| Use of services | Ever used one or more support service | 21 (44) |
|  | Never used any support service | 27 (56) |
| Psychological distress | Met GHQ^a^ cut-off score (≥4) | 26 (62) |

^a^GHQ: General Health Questionnaire.

Table 2: *Workplace support services used by participants*

| **Type of support service (self-reported as used by participants)** | **Number of participants who used this service* (%)** |
| --- | --- |
| Individual counselling | 13 (27) |
| Wellbeing smartphone applications | 4 (8) |
| Wellbeing hub | 4 (8) |
| Occupational therapy | 3 (6) |
| Mindfulness sessions | 2 (4) |
| Psychoeducational sessions | 2 (4) |
| Free food | 2 (4) |
| Q&A/ reflection sessions with management | 2 (4) |
| Free car parking | 1 (2) |

*some participants used more than one service, and some may not have used any, so % do not add up to 100.

# **Appendix 3** – Additional quotes

Socio-political Context Barriers

| Sub-theme | Quote | Participant |
| --- | --- | --- |
| Stigma | It doesn’t matter who you are or what your background or what your circumstances anybody can be affected, and I think that term still, mental illness, mental health, it still has a way to go to be broken down to people feeling happy to talk about it. | Nicky |
|  | Working for an NHS Trust that supports people with mental health - it’s harder for colleagues to speak about their own mental health. The stigma is you don’t want to be seen as weak. You support people with mental health problems but yet you are having a breakdown. | Shay |
|  | I think there’s a general stigma to mental health issues full stop. | Kirstin |
|  | I know this shouldn’t be the case but mental health problems, they have a stigma and it’s really tough for someone to come out and say ‘I’m struggling just to come out of bed every day, I’m struggling to go to work and come back from work’. So, for me I’d rather deal with it in my own way than let them know I’m struggling. I don’t know but it’s related to the stigma, my shame, I guess. | Ahmed |
|  | I think some people sometimes, you can feel a bit embarrassed to get counselling, and I don't know if anything can be done to change that. | Lynne |
|  | I think stigma has always been an issue with mental health, 1 in 4, there’s a lot of stigma with mental health definitely. | Neil |
| NHS under pressure | More than half the staff not available in the office, so it was proper crazy, so we didn’t get a chance at all to even think of anything else. | Brianna |
|  | It was very stressful, we had staff which were scared to work. We had staff that couldn’t work because they had to self-isolate. We had staff that were tested positive. We had agency staff that wouldn’t pick shifts up because they were scared to work on a COVID positive ward. It was down to probably a handful of us, and we burnt out. We were working flat out five, six, seven days of the week. We were working because there was nobody else. | Shay |
|  | There was only three full time nurses and then we were just running on agency and whatever. Generally, you just fire-fought a lot of the time. | Stephen |
|  | I was thinking of retiring at the end of finance year next March, but the way things are possibly coming out now, I think I’m going on my 60th birthday. | Nicky |
|  | I think there is too much work to reduce the hours people are working, even if it’s by an hour every week, because they need to access a service. | Ahmed |

Socio-political Context Enablers

| Sub-theme | Quote | Participant |
| --- | --- | --- |
| Invest in staff and support services | Create a safer staffing model so that there’s enough staff to patients so that you’ve got time to read your emails rather than just constantly running around. | Stephen |
|  | I think they need to deal with the mental health of their staff, because actually you are no good at looking after other people if your personal world is falling apart whether it be directly or indirectly related to work. You can’t be functioning at your best and be there for other people and provide the best care for patients if actually you are not in the best place yourself. | Kirstin |
|  | I think at the beginning obviously it was a little bit difficult because of the volume of it, so many people tried to access it for information… the extent of calls to the hub were way, way over. So, I don’t know, maybe some people did find it difficult to access this information. Or, sorry, not access because they could access it in other ways but to actually speak to somebody. | Ruksanah |
| Normalising help seeking | I think we’ve got more focus on staff wellbeing than what we’ve ever had. | Caroline |
|  | Staff are human we all fail and actually we’ve had a very stressful year and we know need to look after ourselves at a personal level and on an organisational level. | Kirstin |
|  | I think, working through this pandemic, I think it’s given me a lot more patience towards my colleagues, a lot more understanding of other people’s needs, and probably more, what’s the word I’m looking for, I think I’m just probably more mindful of others and how they’re feeling inside. | Shay |
|  | Very good support, I think, from the top downwards who have been saying ‘it’s OK to be not OK and if you need to talk about it you can do’. | Nicky |

Organisational Culture Enablers

| Sub-theme | Quote | Participant |
| --- | --- | --- |
| Visible leadership | The chief executive is doing an all-staff update - click here to basically accept the invite and be part of that. And that’s pretty well, very well attended. | Juliet |
|  | I think from the top down it’s got to be promoted that actually we are looking after our own mental health and making a big deal, that actually if we need to provide support and saying everybody needs it whoever you are, and it’s got to be much more open. | Kirstin |
|  | in terms of the Guardian lady, I did contact her once right at the start of the pandemic and with a few safety concerns, she was really, really helpful actually. And she gets in touch with- she goes to the, the board meetings and raises all these things anonymously for you and then feeds it, feeds it back to you. She did come back to me, and she was really good. | Lynne |
|  | I think it’s important for the chief exec and the executive team to be able to hear from people at ground level on the floor, type of thing because, do say it as it is. | Neil |
|  | Our chief exec, who is male, I think he is actually and we’ve been having weekly bulletins, he’s actually very good about talking about things like that. | Nicky |
| Caring Employer | Just the mental knowing that if I need help it’s going to be there. So that mental assurance was always there. | Brianna |
|  | It’s about feeling like there’s someone out there. It doesn’t necessarily mean that I have to have a conversation with them, or it doesn’t mean that I have to actually do something, but it’s just nice to know the world is out there and some kind of encouragement. | Juliet |
|  | I actually think, having worked for them for a very long time, that actually they have done very well from the support point of view. | Kirstin |
|  | Really, I don’t think our Trust could do anymore. If I was from the outside in, probably like you are doing with all this research, you’d probably think this guy loves this Trust, but for me personally I don’t think they can do any more. | Neil |
|  | I think the Trust has done as much as they can to provide all of this. It’s whether an individual themselves wants to access it or not and how they feel about it. | Ruksanah |
| Supportive Line Management | My senior in my place is very supportive to see what are my needs at the time and how to deal with it. I think the only support I got was from the senior staff who I work with. | Ahmed |
|  | He is very good, very, very supportive. He’s the kind of bloke, if you are poorly, he’ll come to your house to see if you need anything. But he always asks about health first. I think it’s on the supervision, that’s what they ask in general, but he’s always supported me over the years. | Neil |
|  | I am very, very fortunate in having a manager who has obviously got her team’s best interests at heart. | Ruksanah |
|  | After having a good sit down with my manager, and her explaining that a lot of the responsibilities I was trying to take home with me doesn’t lie with me, I have been able to cope with it a bit better. | Warren |
|  | During all our meetings, the huddles we had virtually, they kept on insisting that we go and use them. Our manager is quite good, and they do pass on whatever they know | Brianna |
| Clear communication of support offer | There is now the resilience hub, which is providing more of that, whether that be signposting to services in the community, or it is providing them with information or it is referral on for counselling services or psychological therapy. | Caroline |
|  | On the Trust internet there is a particular site for staff wellbeing and there will be, that’s one thing, there’s a particular button that you can click onto and it brings up a whole lot of resources. | Juliet |
|  | It’s a very good and a very wide-ranging intranet, but it’s like anything, there’s that much there that a lot of the time you become numb to it. | Stephen |
|  | I have every respect for anybody, but say on the cleaning staff, they may not have the opportunity to get onto a computer to see what's available to them, because they’re not working in an office as such - they’re out and about. So, I would imagine stuff like that would find access to digital information much more difficult. | Nicky |
|  | So, for me that can’t walk past anything without reading it yes, I’ll always read posters. | Kirstin |
|  | Sticking up a little poster somewhere hoping that you read it as you walk past the 20,000 other posters. | Shay |
|  | There's been lots of emails about wellbeing stuff, but I've perhaps not read them all…you do, you get inundated with emails, don't you? So, you do tend to ignore them a little bit. | Lynne |
|  | On a daily basis they sent out information of what you could access, where you could go for help and all those kinds of things, and just a constant reminder that there was help out there. | Ruksanah |
|  | So maybe if it just becomes a little bit more promotional, stuff around, especially now, at the moment, that it’s promoted a little bit more than what it actually is. | Jonathan |
|  | I think the big thing is that our 2020 hub phone number is 2020. That tends to be a number that sticks in your head. If all you have to dial is that extension number that’s easy. The fact that it then signposts you to other people is very good. | Kirstin |
|  | You had the staff helpline for the pandemic. | Warren |
| Peer support | It’s a nicer, kind of thing, but it’s more instantaneous, so you can send an email and you have a Teams meeting or something and you might afterwards have a brief chat or a couple of email correspondences, or something about what’s funny about that, or what was dire, or ‘oh my God what do we do next?’ So that’s nice, just a couple of comments, but it doesn’t go into ‘OK let’s sit down and talk about this for two hours’. It’s more instantaneous. And that’s helpful - it just bounces you back a little bit. | Juliet |
|  | I suppose often your colleagues become your counsellor, don't they, in a way…You know, in a good way, in a nice way. We all have a little chat and help each other out and things. | Lynne |
|  | We do have, like I say, peer support. We do look out for each other like I think I’ve already said, like I call the admin lady and I think we do look out for each other. | Neil |
|  | I suppose it’s just offering an ear if somebody wants to get something off their chest whether it be about a work issue, which we’ve had today with one of our members, one of the staff. Just listening to somebody getting it off their chest if they’ve had a bad day. | Nicky |
|  | My whole team have been very, very supportive and I think that’s the only thing that kept us all going, being there for each other. | Ruksanah |
|  | That was one of the things our ward manager wanted to put in place, she introduced the link roles for wellbeing officers, for staff to look out for staff, and for us to have basically not to go home without offloading if there were any concerns or anything we needed to try to deal with and address it there and then. | Shay |
|  | It’s quite informal, it’s not like everything is written down, it’s just an informal catch-up to see how we are all doing. It feels quite supportive as well, it doesn’t feel like a bit of a tick box exercise like some of these can. | Jonathan |
|  | So that was quite nice to be able to chat and go and speak to people from other wards in a COVID safe socially distanced way. | Stephen |

Organisational Culture Barriers

| Sub-theme | Quote | Participant |
| --- | --- | --- |
| Unsupportive employer | But I do feel there’s a political element in it, of there’s pressure on staff to be at work regardless of what they’re going through, and this pandemic has highlighted that. | Caroline |
|  | We think that this is our work, and this is where we should be feeling comfortable and valued, and the fact that you are charging us for coming to work doesn’t make sense. (discussing ending of free parking) | Ahmed |
|  | Things like the management supervision, both giving it and receiving it, it does feel a bit tick box at times because obviously it’s one of the key performance indicators of the Trust. | Stephen |
|  | In NICE guidelines it stipulates that for counselling for an effective counselling treatment is over ten sessions. So, it’s like the NICE guidelines aren’t even being really adhered to is it? So, I just feel that staff shouldn’t be any different than clients and should be treated accordingly…staff are equally as important as clients. | Jonathan |
|  | We didn’t really have a staff room at our hospital. | Stephen |
| Poor line management | I was seeing for a number of years was unacceptable practice from management, and not just the team manager, from senior management. | Caroline |
|  | I know when my manager left a leaflet lying on my desk, I was offended because she'd left it that openly for everyone to see. As if, as if I'd gone to my manager and said, ‘oh no, I need counselling’, and I was embarrassed that she'd left it there. | Lynne |
|  | The only people that can refer is the team manager. Very few people have a referrer, right. I’m sure there possibly are, but as a general rule it’s your manager and I don’t think a lot of managers, I can’t imagine it’s only our Trust, but a lot of people step up into the role when someone else disappears, goes, moves, goes on leave, quits in a sudden blaze of glory, and it’s like, right, you are now acting up as a ward manager, and it’s not like you have the intrinsic knowledge of how you actually go about it. | Stephen |
|  | It could be, I think, for any issues, so you haven’t got to go through the formal process occupational health via your managers and this type of thing, so having that freedom to be able to call somebody, but I think they should carry that on. | Neil |
| Limited Support Available | They do offer something called self-support where you can ring up and make an appointment for counselling, as it were, within the Trust. However, it’s always difficult to get through in general and specifically it’s been almost impossible. | Kirstin |
|  | I think it would be good to offer colleagues more face-to-face meetings rather than giving out wellbeing letters or ‘here’s a phone number ring this up’ and you speak to someone like a robot on the phone. | Shay |
|  | When I needed to access them there was a bit of a waiting list. I think it took, well if I’m going right back to the first contact, I think it took around about a couple of months before I got to see someone. |  |
|  | It feels like an emphasis is obviously put on the clients and even eight sessions that clients are entitled to, that’s really not enough, but for staff only to get five or six, I don’t think that’s right really. I think we should be offered more than that. | Jonathan |
| Staff demoralised | Lack of space, lack of confidentiality. Lack of compassion, lack of respect. Stigmatising, poor leadership. | Caroline |
|  | We lost...seven patients died of coronavirus on the ward, three on one shift, on my shift. I was working with a student nurse, I don’t know if this is irrelevant, but I was working with a student nurse who actually caught coronavirus herself, she beat it and then a few weeks later committed suicide. | Shay |
|  | Individual people don’t want to be made out to be heroes, but it would be nice not to have your pay cut after this shitter of a year. | Stephen |
|  | The sisters meet, and the assistants meet with the sisters, but those of us that are in the middle, we get to be told what’s going on, but actually we don’t have our opinions asked. | Kirstin |
|  | I was thinking of retiring at the end of finance year next March, but the way things are possibly coming out now I think I’m going in October on my 60th birthday. | Nicky |

Individual Experience Barriers

| Sub-theme | Quote | Participant |
| --- | --- | --- |
| Not aware of support | I've not really ever looked into it. | Lynne |
|  | I wouldn’t say I’m fully aware of everything what’s available. It would be helpful if, but then again, I’ve never looked on Connect to see if everything is available on that. | Warren |
|  | No, that is definitely not promoted well enough. I actually stumbled across it, really. | Jonathan |
| Lack of time to access support | There’s a recreational kind of area. There’s armchairs and things like that. Our manager has got two of them in the meetings that we could have availed, but I haven’t had time. | Brianna |
|  | I would be facilitating the leadership meeting and you’d be delivering that information that came top down of what’s available - you would get an eye roll and it’s ‘I haven’t got time to access that’. | Caroline |
|  | I’m thinking why I didn’t access services and most of it literally was down to time. | Juliet |
|  | If you had a break, you generally stayed on the ward. You just find a room that you could lock yourself in for ten minutes or twenty minutes. There regularly wasn’t too much scope to have breaks. | Stephen |
|  | I think the biggest issue, like we touched on earlier, is just the time it takes to access them, and to be able to attend them during works time. I think that’s possibly always going to be a stumbling block, | Warren |
| Bad experience of support service | My experience within the Trust has been, I have been let down in the past and confidentiality has been breached when I’ve sough support. Therefore, the trust isn’t there for me to go forward for a therapy service. | Caroline |
|  | We’ve got the occupational therapy which is - they’re a third sector agency. My personal experience of them is they’re terrible. | Stephen |
|  | There was a group I accessed after let’s talk wellbeing and I found the whole experience very uncomfortable, and I remember colleagues, one of my other colleagues felt that as well. I just wish I could remember the name of it. It was like they didn’t really have time for you, felt very cold and wasn’t very approachable during the sessions. | Warren |
|  | The other day they had a wellbeing thing, and they were talking about facemasks and stuff like that and, I don’t know, being a bit of an alpha male I’m not going to be going to have a face mask or anything like that am I? | Jonathan |
|  | I know from past experience, with the internal one you can actually have come out the other side of whatever it was you wanted support for before you actually get your appointment. | Kirstin |
| Toxic Stoicism | I could read some online resources, or I could have a chat with someone, but it probably just boils down to time at the end of it and my decision to say OK I can struggle on for a bit longer, get over it. | Juliet |
|  | You're seen as a, perhaps a stronger person if you don't need it. | Lynne |
|  | Yes, we just went through it… thinking about it I don’t know how everyone coped now. | Brianna |
|  | Well, I think during the time here it would be considered a waste of time. If somebody needs it, they have to access it out of hours. During work hours we’re short of people. | Ahmed |
|  | It’s still a place of work. It’s not a holiday club. | Neil |
|  | I wanted to, but I didn’t have the time. I don’t know what it’s like where you are but trying to access the GP is like winning the lottery. | Shay |
|  | We’re quite limited with our Trust, to be honest, and again, not that I don’t, it’s not like I took a job as a nurse expecting to be showered with gifts and opportunities, it’s not that sort of job to be honest. | Stephen |
|  | If it could be accessed as quick as a few days, then fantastic, but I don’t think that would be the case. It’s like everything, isn’t it, in healthcare - unfortunately there’s always a waiting list. | Warren |
